# Diagnostic and Therapeutic Challenges of Comorbid ASD, ADHD and Psychosis: A Case Report

**DOI:** 10.3390/bs12100382

**Published:** 2022-10-06

**Authors:** Veronica Scarselli, Melania Martucci, Maria Novelli, Serena Galosi, Maria Romani, Carla Sogos

**Affiliations:** Department of Human Neurosciences, Sapienza University of Rome, 00185 Rome, Italy

**Keywords:** Autism, early onset psychosis, ADHD

## Abstract

Autism Spectrum Disorder (ASD) and attention deficit hyperactivity disorder (ADHD) comorbidity is common in clinical practice and it seems to be related to shared etiological mechanisms and genetic susceptibility. Moreover, occurrence of psychosis can further complicate these complex clinical pictures. Here, we discuss the case of a nine-years-old boy presenting with an episode of abnormal sustained posture of the upper limbs, resembling dystonia, at the age of 3. At this time, auditory and visual hallucinations, as well as obsessive thoughts and attentional lability were also present and a diagnosis of “Early onset psychosis” was initially made. Due to the worsening of clinical picture, several hospitalizations were necessary and pharmacological treatment with carbamazepine, risperidone and aripiprazole was carried out. Extensive clinic evaluation revealed a past medical and personal history of toe walking, weak social skills and stereotyped behavior observed and ADOS-2 Module 2 administration revealed severe Autism scores. Moreover, signs of attention and hyperactivity were consistent with ADHD diagnosis. This work highlights the importance of a complete diagnostic assessment in patients with complex presentation, suggesting the possible overlap diagnosis of ADHD and Autism even in presence of psychotic-like symptoms.

## 1. Introduction

A high rate of multiple comorbidities in neurodevelopmental disorders is a common finding [1]. Between 37–85% of Autism Spectrum Disorder (ASD) are estimated to present comorbid attention deficit hyperactivity disorder (ADHD) symptoms, with amplification of negative impact on quality of life [2,3]. Moreover, psychotic disorders may further complicate these clinical pictures, resulting in diagnostic and therapeutic challenges for pediatric psychiatrists. We should consider that ASD and schizophrenia are both characterized by atypical neuro-development of language and difficulties with social interaction and communication and ASD symptoms appear to overlap most significantly with negative symptoms of schizophrenia (see Table 1) [4]. For example, difficulties with emotional reciprocity, or speech delay observed in ASD may be understood in terms of blunting of affect or alogia (poverty of speech) in schizophrenia, respectively [5]. Catatonic features may also be present in both disorders. Conversely, positive symptoms, such as hallucinations and delusions, are defining features of schizophrenia but are not typically symptomatic of ASD [5]. Age of onset constitutes another significant distinction between the two constructs [6]. To date, little is known about mutual interactions between neurodevelopmental disorders, and the influence of psychosis on them. The research on neuropsychological processes has mainly revolved around executive function (EF) and theory of mind (ToM) [7,8], emphasizing the presence of common etiological mechanisms and shared genetic risk factors [9,10]. A recent metanalysis suggested an increased risk of psychotic disorder (PD), with a pooled relative effect of 4.74 (95% CI, 4.11–5.46) for patients with childhood ADHD. The association remained high for the more restrictive diagnosis of schizophrenia. The association between ADHD and subsequent psychosis could be explained considering many potential mechanisms, including shared genetic susceptibility or social environmental factors [11]. Furthermore, prenatal factors, such as diabetes during pregnancy or neonatal complications, are also frequently reported as risk factors for both disorders. It is also possible that the association between ADHD and PD is mediated by other factors. Attention-deficit/hyperactivity disorder has been described as a risk factor for substance use disorder (SUD), possibly because of increased impulsivity and a deficit in the dopaminergic reward system, and SUD, especially the use of cannabis, has been described as a risk factor for PD [12,13]. Krowkoski et al. suggested that a combined ASD–ADHD phenotype is characterized by two latent ASD domains (social communication and repetitive restrictive behavior) and two latent ADHD domains (inattention and hyperactivity/impulsivity), as demonstrated by an exploratory factor analysis and a confirmatory factor analysis [14]. Moreover, some evidence suggests three separate pathways explaining the comorbidity between ADHD and ASD. These pathways are from impulsivity to social information processing difficulties, from hyperactivity to restricted and repetitive behaviors and a pairwise pathway between inattention, verbal IQ and social information processing difficulties [15]. These data and others have led some to opine that it is not possible to determine if ADHD symptoms in ASD represent ASD, comorbid ADHD or a separate condition entirely. At this point, clinical impression remains the deciding factor which determines the most appropriate diagnosis for a patient (see Table 1) [8]. In this context, we discuss an emblematic case of a nine-year-old boy presenting with complex clinical features and overlapping conditions, requiring extensive and accurate evaluation.

## 2. Case Presentation

### 2.1. Social and Family History

The patient, Z, now a nine-year-old boy, was born to non-consanguineous parents. His father presented delusions of jealousy and persecution from the age of 20 years. Moreover, a maternal aunt suffered from epilepsy and other relatives in the maternal line were treated for unspecified anxiety and psychotic disorder. Finally, familiarity for intellectual disability in maternal and paternal lines was also reported.

### 2.2. Personal and Development History

The mother was primiparous and she denied consuming ethanol or illicit drugs during pregnancy. Z was born at term without complications and he did not demonstrate postnatal issues. Walking and talking milestones were in the normal range, however, a tendency to toe walking was reported. Moreover, parents reported from the first year of life, exaggerated responses to environmental stimuli (loud noises) and from the second year, of life repetitive behaviors such as turning on/off lights. At the age of 3 years, he presented with an acute episode of abnormal posture of the upper limbs maintained for about 10 h resembling dystonia. He was taken to the emergency room and later admitted to a hospital and a first diagnostic suspicion of chorea was made. Abnormal postures regressed spontaneously, however, after a few months, the clinical picture rapidly worsened; at this time, he was referred to our tertiary care center with concerns about marked irritability, obsessive thoughts, persistent impairment due to inattention. Moreover, auditory and visual hallucinations were reported.

### 2.3. Physical and Neurological Examination

The patient looked to be his stated age and had an average body build. He did not have dysmorphic facial features and neurological examination was unremarkable. At the time of our consultation, his interactions with the examiner were somewhat elusive; eye contact as well as communication skills were poor. He also exhibited repetitive patterns of behavior, interests and activities and he was impulsive and hyperactive. He presented rituals related to bathing and hand washing that were extremely disabling.

### 2.4. Laboratory Tests, Imaging and Genetic Assessment

Blood counts, renal and liver function tests, thyroid profile and other laboratory tests were unremarkable. In the suspicion of immune-mediated, infectious and/or metabolic disease, due to the aggravation of the clinical picture, the following examinations were also performed and resulted negative: metabolic work up, CSF analysis, serum autoantibodies. Moreover, an electroencephalogram (EEG) was performed and revealed nonspecific cerebral dysfunction and no epileptiform discharges. Finally, a brain magnetic resonance (MRI) showed no abnormalities. An array comparative genomic hybridization (aCGH) was performed on the patient’s parents and revealed normal genes. The FMR1 genetic test was likewise negative. He is now a candidate to whole-exome sequencing (WES).

### 2.5. Diagnosis

Even though an initial suspicion of chorea and early onset psychosis was posed, owing to the peculiar personal and developmental history, an in-depth diagnostic evaluation was performed. The Kiddie Schedule for Affective Disorders and Schizophrenia for School-Age Children Mania Rating Scale for Children and Adolescents [17] was administered to parents and confirmed a clinical score for Autism and ADHD. Due to deficits in social communication and repetitive patterns of interests, an ADOS-2 (Autism Diagnostic Observation Schedule) [18] was administered, reporting a total score of 13, which therefore exceeds the Autism cut-off in the ADOS-2 classification. An Autism diagnosis, according to DSM 5 criteria, ADOS2 and ADI-r scores [19], was made. The full assessment also included a cognitive evaluation and other specific tests exploring executive functions and psychiatric issues (i.e., the Nepsy II, Tower of London, CY-BOCS). Cognitive evaluation (Wechsler Intelligence Scale for Children-IV) [20] at 8 showed a full scale IQ score of 80, with a major discrepancy between the Verbal Comprehension Index (VCI = 106) and the Elaboration Speed Index (ESI = 53). Despite FIQ indicating low scores, cognitive functioning appears to be higher than measured and marred during the assessment by extreme hyperactivity and inattention. The Child Behavior Checklist [21] and the Conners’ Rating Scales-Revised [22] were administered to the parents and confirmed a clinical score for ADHD. Hence, he was found to meet the DSM-5 criteria for attention-deficit/hyperactivity disorder (ADHD) (combined-type). The Nepsy II [20] test revealed immature performance in understanding mental constructs, beliefs, deceptions and fictions (Theory of the Mind part A) as well as poor ability to set shift, highlighting an impulsive response style with poorly controlled output, poor cognitive flexibility, poor planning and adherence to rules. The Tower of London test [23] showed immature higher-order capabilities such as planning, strategic decision making and effective execution. Finally, mild obsessive-compulsive symptoms emerged from the Children’s Yale-Brown Obsessive Compulsive Scale (total score 11, Obsessions = 11, Compulsions = 0) [24]. The goal of this tool has been to assess the severity and quality of OC symptoms.

### 2.6. Management

Z initially received psychomotor and speech therapy, group therapy and at-home educational support. For the subsequent years, the patient was being followed up by occupational, behavioral and speech therapists. He has shown mild improvement in his attention span and communication skills. Despite dedicated work in behavioral and speech therapy, he is still showing dysfunctional behavior and he was not able to achieve a good development of adaptive and daily-living skills. Now he is attending primary school with a support teacher.

As most of ADHD plus ASD patients, Z required a combination of medication, psychosocial and behavioral interventions. For initial pharmacological management of emotional lability, carbamazepine was prescribed in another hospital, and then increased to 20 mL/die without significant clinical improvement on behavioral troubles. Subsequently, due to the high rate of irritability, risperidone was prescribed by our center, but rapidly discontinued due to the appearance of gynecomastia and hyperprolactinemia. Then, aripiprazole was titrated up to 10 mg/die with improvement in his behavior as well as him being calmer and more responsive. At this regard, the evidence confirms that the second-generation antipsychotics, particularly risperidone and aripiprazole, have been used for the short-term treatment of angry outbursts in children and adolescents with ASD, some of whom will have ADHD [25]. Low doses, regular reviews and short-term use are advised. However, there are variable degrees of official approval for their use between countries and behavioral and environmental interventions are first-line treatments [25]. The improvement of the symptomatology was confirmed by using the Clinical Global Impression Scale [26]. The patient’s score at admission to the project (CGI-S) was 6 (much worse). After the treatment, the patient’s score (CGI-I) was 5 (minimally worse).

## 3. Discussion

We reported a complex case of a nine-year-old boy who, crossing habitual diagnostic categories, presented with psychotic features and was finally diagnosed with ADHD and ASD. This case is emblematic of the myriad challenges of diagnosis in neuropsychiatric disorders and encourages careful evaluations in children presenting with multiple atypical symptoms. Actually, it is known that certain clinical features, such as psychomotor disturbances, social withdrawal and poor communication, stereotypies and perceptual disturbances, are characteristics of both psychotic disorders and ASD [27,28].

Moreover, the presence of auditory hallucinations in ASD has been reported [29].

Furthermore, a high rate of ADHD/ASD comorbidity is now established, and this dual diagnosis is often made, since DMS5 allows comorbid diagnosis [15].

Our patient presented psychotic features at onset, but his personal history analysis revealed features attributable to Autism Spectrum Disorder. What is more, the rapid evolution of the clinical picture was characterized by evidence of marked motor hyperkinesia and difficulty in concentration, compatible with ADHD. In this context, psychotic features resulted as the first manifestation and part of a complex clinical picture characterized by developmental disorders in a complex family environment.

Regarding the overlap diagnosis of ASD and ADHD, some authors suggest they are different manifestations of one overarching disorder, in which emotion regulation is a crucial common factor [30].

However, multiple executive functioning impairments are associated with both ADHD and ASD [31] and recent research has identified several neurocognitive features and underlying genetic substrate [32] that could help explain the co-occurrence of ADHD and Autism. However, neuropsychological assessment of executive functions does not fully account for the complexity of symptoms, so that clinical data and prolonged observation are essential for diagnosis.

This work highlights the importance of a complete diagnostic assessment in patients with unusual and complex semiological presentation [33], suggesting the possible overlap diagnosis of ADHD and Autism even in presence of psychotic-like symptoms.

## 4. Conclusions

The high prevalence of mental health and neurodevelopmental disorders comorbidities requires an accurate psychological assessment and use of standardized assessments and a diagnostic and statistical manual of mental disorders (DSM)-5-based assessment.

It can be useful to detect specific underlying processes differentiating the comorbid syndrome and plan an appropriate management and rehabilitation program.

## Figures and Tables

**Table 1:** Overlap symptoms and differences between ADHD, ASD and psychosis.

Overlap Symptoms	Differences
Inattention (ASD + ADHD) [15]	Restricted interests (ASD) [16]
Problems in social interaction with peers (ASD + ADHD) [14]	Restricted repetitive behavior (ASD) [16]
Atypical neuro-development of language (ASD + Psychosis) [5]	Impulsivity (ADHD) [16]
Difficulties with social interaction and communication (ASD + Psychosis) [7]	Evidence of prominent hallucinations for at least one month (Psychosis) [16]
Impairment in social functioning (ASD + Psychosis) [7]	Evidence of prominent delusions for at least one month (Psychosis) [16]
Difficulties with emotional reciprocity (ASD + Psychosis) [6]	Struggle with inhibition (ADHD) [1]
Speech delay or absence/alogia (ASD + Psychosis) [6]	Struggle with planning (ADHD) [1]
Catatonic features (ASD + Psychosis) [6]	Struggle with cognitive flexibility (ASD) [1]

## Data Availability

Not applicable.
